# Visceral adiposity index is associated with lung function impairment: a population-based study

**DOI:** 10.1186/s12931-020-01599-3

**Published:** 2021-01-06

**Authors:** Sunyue He, Jie Yang, Xiaoyong Li, Hongxia Gu, Qing Su, Li Qin

**Affiliations:** 1 Department of Endocrinology, Xinhua Hospital Chongming Branch, School of Medicine, Shanghai Jiaotong University, 25 Nanmen Road, Shanghai, China; 2 Department of Endocrinology, Xinhua Hospital, School of Medicine, Shanghai Jiaotong University, 1665 Kongjiang Road, Shanghai, 200092 China

**Keywords:** Lung function, Visceral adiposity index, Visceral adiposity

## Abstract

**Background:**

The effects of visceral adiposity on decreased lung function have drawn much attention. Recently, the visceral adiposity index (VAI) has been proposed as a visceral fat distribution and dysfunction marker. However, the relationship between the VAI and lung function has not been investigated. The objective of the study was to analyze the association between the VAI and lung function and evaluate the potential of VAI as a predictor of lung function.

**Methods:**

We collected data from a population-based study of 1786 subjects aged 40 years or older. All subjects completed a questionnaire and underwent anthropometric measurements and laboratory tests. Linear and logistic regression models were developed to assess the association between the VAI and lung function.

**Results:**

The VAI was inversely related to FVC%predicted in men and negatively associated with both FVC%predicted and FEV1%predicted in women. In the linear regression analysis, the decrease in FVC%predicted associated with each 10% increase in the VAI was 1.127% in men and 1.943% in women; the decrease in FEV1%predicted associated with each 10%increase in the VAI was 0.663% in men and 1.738% in women. Further regression analysis revealed that the VAI was positively correlated with FVC and FEV1 impairment in women.

**Conclusions:**

We were the first to show a clear correlation between the VAI and lung function impairment in the Chinese population. The VAI could be a simple and reliable approach in daily practice, and individuals, especially women with a high VAI, should receive additional screening and preventive interventions for respiratory disease.

## Introduction

Impaired pulmonary function is a predictor of mortality related to various diseases independent of diagnosed lung disease and smoking status [1]. However, there is less information on the determinants of lung function other than cigarette smoking in the general population. Previous studies have suggested that obesity is inversely associated with respiratory functions, including a reduction in forced expiratory volume in the first second and forced vital capacity. In a large-scale population-based study, abdominal obesity was the key determinant of the association between decreased lung function and metabolic syndrome, independent of major cardiovascular risk factors [2]. Since waist circumference (WC) is a great indicator of abdominal obesity, it is correlated with both subcutaneous fat and visceral fat, which contribute to the relationship between abdominal obesity and lung function, respectively [3–6]. A recent study noted that in subjects with abdominal obesity, the main determinant of lung function impairment is the presence of visceral fat [7].

Advanced and accurate measuring techniques such as computed tomography and magnetic resonance imaging (MRI) are considered the gold standard for the quantitative evaluation of visceral adipose tissue (VAT) [8]. However, these techniques result in radiation exposure, are time consuming and costly are not always feasible, particularly in large-scale population studies. Recently, the visceral adiposity index (VAI) has proven to be a marker of both visceral fat distribution and dysfunction [9]. The VAI provides a sex-specific estimate of risk based on WC, body mass index (BMI), serum triglycerides (TGs) and high-density lipoprotein cholesterol (HDL-c) [10]. Subsequent studies confirmed correlations of the VAI with visceral fat distribution and insulin resistance, even in patients with a normal WC. The VAI has been applied in different patient populations, including patients with metabolic syndrome [11, 12], diabetes [13], nonalcoholic fatty liver disease [14] and cardiovascular disease [15, 16], all of which are often associated with lung function impairment [17–19]. There is a lack of data on the association between VAI and lung function impairment. Therefore, the objective of the present study was to explore the possible impact of the VAI on lung function in a large population-based Chinese middle-aged and elderly sample and to address whether VAI can replace visceral fat measurement for clinical screening.

## Materials and methods

### Study population

This study is a part of the Risk Evaluation of Cancer in Chinese Diabetic Individuals: a longitudinal (REACTION) study, which was a community study conducted among adults aged 40 years and older. The study design and methods have been described previously in detail. The data presented here are based on the baseline survey of subsamples from the Shanghai Chongming District in eastern China. A total of 10,060 eligible subjects participated in the research. Individuals meeting the following criteria were excluded: (1) those with missing data for WC, height, body weight, blood pressure and metabolic variables and (2) patients with a history of chronic lung diseases, self-reported asthma or malignant diseases. Thus, a total of 8840 participants were eventually included in this analysis. A total of 1786 participants received a lung function test and were eventually included in the analysis.

### Data collection

Essential information on demographic characteristics (e.g., age, sex, ethnicity, education), smoking status, habit of alcohol consumption, and disease history were collected by interviews conducted by certified medical workers using a standardized questionnaire. WC was measured at the midpoint between the lowest costal margin and the lateral iliac crest. Systolic and diastolic blood pressure (SBP and DBP) recordings were measured three times from the right arm of patients in a sitting position after 30 min of rest. The measurements were taken in 5 min intervals, and a mean value was used in the statistical analysis. BMI was calculated as weight in kilograms divided by the square of height in meters. Education was divided into five categories. Smoking status was divided into two categories: current nonsmokers and smokers who smoke occasionally or every day. The habit of alcohol consumption was divided into two categories: current nondrinkers and drinkers who drink occasionally or every week.

After an overnight fast of at least 8 h, venous blood samples were collected for the measurement of the levels of various factors, including serum fasting plasma glucose (FPG), hemoglobin A1c (HbA1c), insulin, HDL-c, low-density lipoprotein cholesterol (LDL-c), TGs, total cholesterol (TC), creatinine and uric acid. Plasma glucose levels were tested by the glucose oxidase method (ADVIA-1650 Chemistry System, Bayer, Leverkusen, Germany). Fasting insulin was measured by radioimmunoassay (RIA) (Linco Research, St. Charles, MO). HbA1c was assessed by high-performance liquid chromatography (BIO-RAD, D10, CA). HDL-c, LDL-c, TGs, uric acid (UA), and serum creatinine (SCr) were measured in fasting blood samples using an automated biochemical instrument (Coulter UniCel DxC 800, Beckman, Miami, FL, USA). Glomerular filtration rate (GFR) was estimated using the equation described by Liu et al. [20].

The VAI was calculated by the following sex-specific equation [10], where WC is expressed in cm and TGs and HDL-c are expressed in mmol/L. Men: VAI = $\left(\frac{\mathrm{WC}}{39.68+\left(1.88\times \mathrm{BMI}\right)}\right)\times \left(\frac{\mathrm{TG}}{1.03}\right)\times \left(\frac{1.31}{\mathrm{HDL}-\mathrm{c}}\right)$; Women: VAI = $\left(\frac{\mathrm{WC}}{36.58+\left(1.89\times \mathrm{BMI}\right)}\right)\times \left(\frac{\mathrm{TG}}{0.81}\right)\times \left(\frac{1.52}{\mathrm{HDL}-\mathrm{c}}\right)$.

### Lung function measurements

Lung function tests, including forced vital capacity (FVC) and forced expiratory volume in 1 s (FEV1), were conducted by a trained physician using an electronic spirometer (Model BF-II, Jintan, China). Each participant was in a seated position with nose clips in place. First, the participant breathed normally and then exhaled steadily into the mouthpiece for as long as possible after full inspiration. The maximum value was recorded after at least acceptable maneuver times. The predicted values for FVC and FEV1 were calculated from the following equations obtained from a representative sample of the Chinese population. Age is expressed in years, height is expressed in cm, and weight is expressed in kg [18].

$$\mathrm{Predicted FVC of men}=-4.33058-\left(0.01326\times \mathrm{age}\right)+\left(0.04669\times \mathrm{height}\right)+\left(0.01664\times \mathrm{weight}\right)$$

$$\mathrm{Predicted FVC of women}=-4.79287-\left(0.01326\times \mathrm{age}\right)+\left(0.04669\times \mathrm{height}\right)+\left(0.01664\times \mathrm{weight}\right)$$

$$\mathrm{Predicted FEV}1\mathrm{ of men}=-3.65523-\left(0.01850\times \mathrm{age}\right)+\left(0.04283\times \mathrm{height}\right)+\left(0.009228832\times \mathrm{weight}\right)$$

$$\mathrm{Predicted FEV}1\mathrm{ of women}=-4.04947-\left(0.01850\times \mathrm{age}\right)+\left(0.04283\times \mathrm{height}\right)+\left(0.009228832\times \mathrm{weight}\right)$$

The percentage of predicted values for FVC (FVC%predicted) is equal to FEV1 divided by the predicted values of FVC. The percentage of predicted values for FEV1 (FEV1%predicted) is equal to FEV1 divided by the predicted values of FEV1. The ratio of FEV1 to FVC was calculated. FVC%predicted < 80% is considered FVC impairment, and FEV1%predicted < 80% is considered FEV1 impairment.

### Statistical analysis

Continuous variables are presented as the means ± standard deviations or medians (interquartile ranges), and categorical variables are reported as numbers (percentages). The normality of variables was evaluated by the Kolmogorov–Smirnov test. Initial analyses showed that the influence of VAI on lung function differed between men and women; therefore, subsequent analyses were performed for men and women separately. Comparisons of continuous variables between the two groups were performed using unpaired Student’s t-test, and comparisons between multiple groups were performed using one-way ANOVA. Partial Spearman’s correlations were performed to evaluate the associations between various related parameters and lung function. Variables associated with lung function in partial Spearman’s correlation analysis were further entered in multiple stepwise linear regression to identify the independent predictive factors of lung function. To avoid the effect of collinearity of the VAI and TGs, TGs were not included in the multiple stepwise linear regression. In addition, we used a logistic regression model to estimate the association between the VAI and lung function impairment. Educational level, smoking status, drinking habit and factors associated with lung function in partial Spearman’s correlation analysis but not included in the VAI equation, including age, blood pressure, FPG, TC, uric acid, and eGFR, were considered potential confounding variables and adjusted in the regression models. All statistical analyses were performed with SPSS 22.0 (SPSS Inc.; Chicago, IL). Every analysis was two-tailed, and a P value < 0.05 was considered to indicate statistical significance.

## Results

### Demographic and clinical characteristics of the study population

The total of 1786 participants aged 40–79 years were included, with 649 men and 1137 women. Participant characteristics are summarized by sex in Table 1. Men and women differed significantly in age, lifestyle factors such as smoking and drinking habits, WC, blood pressure, FPG, HDL-c, LDL-c, TC, and UA. The median VAI was 1.96 ± 1.52 in men and 2.48 ± 1.65 in women. Men had higher frequencies of smoking and drinking and lower mean values of FVC%predicted and FEV1%predicted than did women. FVC%predicted and FEV1% predicted were 84.13 ± 14.69% and 85.18 ± 16.81% in men and 86.93 ± 15.73% and 93.20 ± 17.95% in women, respectively. Additionally, the average FEV1/FVC in men and women was 80.66 ± 9.38% and 83.98 ± 7.37%, respectively.

**Table 1:** Demographic and clinical characteristics of the study population

	Men (649)	Women (1137)	P value
Age	57.39 ± 7.64	55.17 ± 8.02	P < 0.001
Smoking	328 (50.5%)	20 (1.8%)	P < 0.001
Drinking	320 (49.3%)	66 (5.8%)	P < 0.001
BMI	24.77 ± 3.25	24.82 ± 3.70	P = 0.793
WC	87.97 ± 9.16	84.02 ± 10.62	P < 0.001
Heart rate	76.91 ± 12.03	81.44 ± 11.54	P < 0.001
SBP	137.96 ± 19.18	133.24 ± 18.73	P < 0.001
DBP	84.18 ± 10.27	81.14 ± 10.10	P < 0.001
FPG	6.58 ± 1.90	6.33 ± 1.62	P = 0.003
HbA1c	5.79 ± 1.03	5.79 ± 0.99	P = 0.958
Insulin	6.96 ± 9.64	7.60 ± 4.05	P = 0.052
TGs	1.41 (0.99–2.17)	1.41 (1.00–2.02)	P = 0.637
HDL-c	1.28 ± 0.33	1.36 ± 0.29	P < 0.001
LDL-c	2.67 ± 0.70	2.82 ± 0.73	P < 0.001
TC	4.84 ± 0.86	5.05 ± 0.90	P < 0.001
VAI	1.96 ± 1.52	2.48 ± 1.65	P < 0.001
Uric acid	0.296 ± 0.069	0.232 ± 0.062	P < 0.001
Creatine	77.11 ± 15.92	63.34 ± 8.45	P < 0.001
eGFR	117.80 ± 24.64	122.18 ± 21.35	P < 0.001
FVC liter	3.22 ± 0.65	2.39 ± 0.47	P < 0.001
FVC%pred	84.13 ± 14.69	86.93 ± 15.73	P < 0.001
FEV liter	2.60 ± 0.60	2.01 ± 0.40	P < 0.001
FEV1%pred	85.18 ± 16.81	93.20 ± 17.95	P < 0.001
FEV1/FVC	80.66 ± 9.38	83.98 ± 7.37	P < 0.001

The values are presented as the mean ± standard deviation (median with interquartile range) or number (proportions)

*BMI* body mass index, *WC* waist circumference, *FPG* fasting plasma glucose, *HbA1c* hemoglobin A1c, *DBP* diastolic blood pressure, *SBP* systolic blood pressure, *HDL‐c* high‐density lipoprotein cholesterol, *LDL‐c* low‐density lipoprotein cholesterol, *TC* total cholesterol, *TGs* triglycerides, *VAI* visceral adiposity index, *eGFR* estimated glomerular filtration rate, *FVC* forced vital capacity, *FEV1* forced expiratory volume in 1 s

### FVC%predicted and FEV1%predicted in different quartiles of VAI

**Fig. 1:**
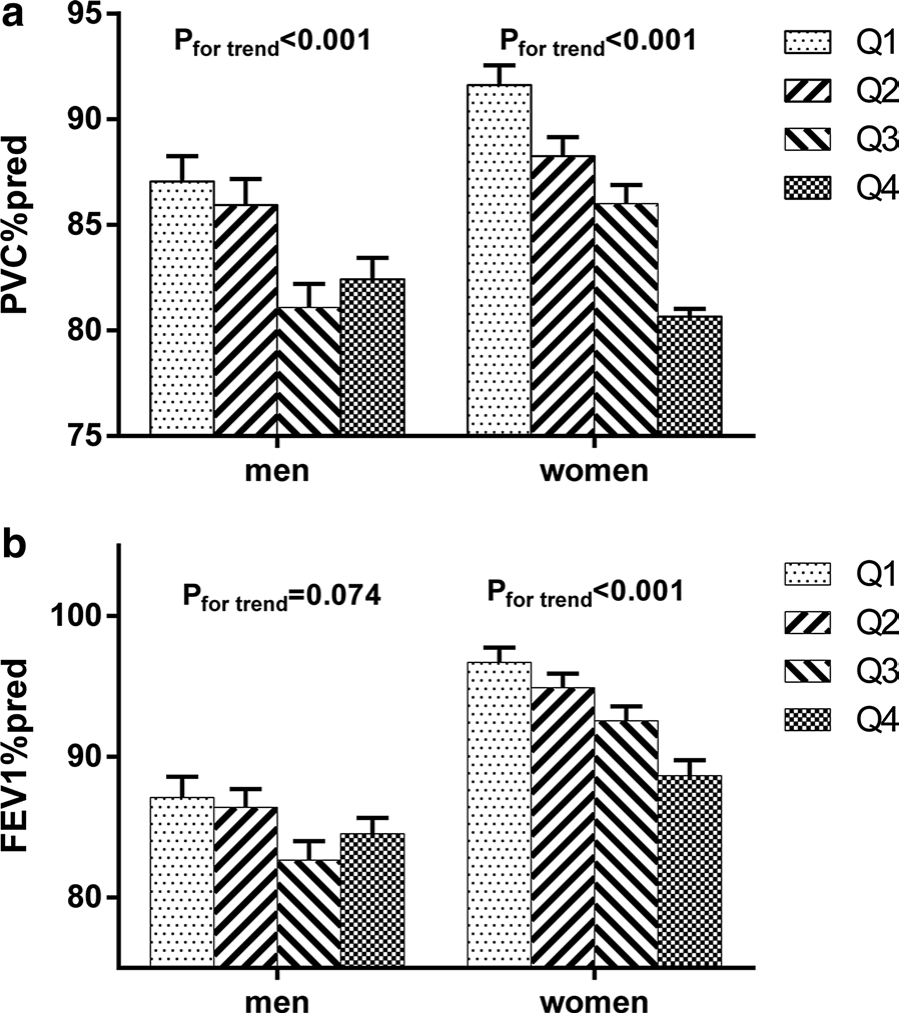
FVC%predicted and FEV1%predicted in different quartiles of VAI. Data are shown as means ± standard error of means

From the lowest to the highest quartile of VAI, the mean values of FVC%predicted were 87.05 ± 1.20, 85.94 ± 1.23, 81.10 ± 1.11 and 82.43 ± 1.01 in men (P for trend < 0.001) and 91.62 ± 0.94, 88.26 ± 0.89, 86.01 ± 0.88 and 80.66 ± 0.37 in women (P for trend < 0.001) (Fig. 1, Table 2). Strikingly, a greater FVC%predicted decrease corresponding with VAI increase was observed in women than in men. The same trend of FEV1%predicted was observed in women; FEV1%predicted decreased from 96.71 ± 1.05 to 94.91 ± 1.00, 92.56 ± 1.03 and 88.66 ± 1.11 as VAI quartiles increased. However, there was no significant difference between FEV1%predicted according to VAI quartiles in men (P for trend = 0.074).

**Table 2:** Lung function in different quartiles of VAI

Men					
VAI	Q1 (163)	Q2 (162)	Q3 (163)	Q4 (161)	P value
FVC liter	3.18 ± 0.67	3.27 ± 0.73	3.18 ± 0.63	3.26 ± 0.58	P = 0.407
FVC%pred	87.05 ± 1.20	85.94 ± 1.23	81.10 ± 1.11	82.43 ± 1.01	P < 0.001
FEV1 liter	2.55 ± 0.64	2.62 ± 0.63	2.58 ± 0.60	2.66 ± 0.51	P = 0.33
FEV1%pred	87.12 ± 1.47	86.41 ± 1.29	82.67 ± 1.35	84.54 ± 1.13	P = 0.074
FEV/FVC (%)	79.79 ± 0.84	80.30 ± 0.75	80.92 ± 0.71	81.65 ± 0.63	P = 0.316

Women					
VAI	Q1 (284)	Q2 (279)	Q3 (290)	Q4 (284)	P value
FVC liter	2.48 ± 0.47	2.42 ± 0.48	2.39 ± 0.43	2.28 ± 0.47	P < 0.001
FVC%pred	91.62 ± 0.94	88.26 ± 0.89	86.01 ± 0.88	80.66 ± 0.37	P < 0.001
FEV1 liter	2.09 ± 0.41	2.04 ± 0.41	1.99 ± 0.36	1.91 ± 0.42	P < 0.001
FEV1%pred	96.71 ± 1.05	94.91 ± 1.00	92.56 ± 1.03	88.66 ± 1.11	P < 0.001
FEV1/FVC (%)	84.17 ± 0.42	84.17 ± 0.40	83.95 ± 0.51	83.98 ± 0.22	P = 0.819

The values of FVC%pred and FEV1%pred are shown as the means ± standard error of means

*VAI* visceral adiposity index, *eGFR* estimated glomerular filtration rate, *FVC* forced vital capacity, *FEV1* forced expiratory volume in 1 s

### Association of the VAI with impaired lung function

Tables 3 and 4 show the correlation coefficients of lung function with the VAI and other metabolic variables. Pearson’s correlation analyses revealed that VAI, WC, BMI, age, SBP, FBG, HDL-c, UA and eGFR were significantly correlated with FVC%predicted in men, and VAI, WC, BMI, age, SBP, DBP, FBG, HDL, TC and UA were significantly correlated with FVC%predicted in women. However, after performing multivariate stepwise linear regression analysis, we found that VAI (β = − 0.098, 0.001) was only negatively and significantly correlated with FVC %predicted in addition to WC and SBP in women. Similarly, FEV1%predicted was negatively associated with VAI, WC, BMI, age, SBP, DBP, FBG, HDL-c and UA. Only VAI (β = − 0.097, 0.002), age (− 0.137, < 0.001), WC (− 0.217, < 0.001), and SBP (− 0.072, 0.025) in women were independent factors in the multivariable stepwise linear regression analysis. However, because BMI and WC were highly correlated (r = 0.783 in men, r = 0.744 in women) and the relationship between WC and lung function was stronger than that between BMI and lung function, BMI was ultimately not included in the multiple stepwise linear regression. In addition, in the linear regression analysis, the decrease in FVC%predicted associated with each 10% increase in the VAI was 1.127% in men and 1.943% in women; the decrease in FEV1%predicted associated with each 10% increase in the VAI was 0.663% in men and 1.738% in women (data not shown).

**Table 3:** Factors associated with FVC%predicted by Pearson’s correlation and multivariable stepwise linear regression analysis

	Men						Women					
	Pearson’s analysis		Stepwise linear regression analysis				Pearson’s analysis		Stepwise linear regression analysis			
	Coefficient	P value	β	SE	t	P value	Coefficient	P value	β	SE	t	P value
VAI	− 0.120	P = 0.001					− 0.219	P < 0.001	− 0.098	0.284	− 3.26	P = 0.001
WC	− 0.368	P < 0.001	− 0.37	0.06	− 10.4	P < 0.001	− 0.351	P < 0.001	− 0.291	0.046	− 9.3	P < 0.001
BMI	− 0.301	P < 0.001					− 0.295	P < 0.001				
Age	− 0.203	P < 0.001	− 0.206	0.069	− 5.76	P < 0.001	− 0.075	P = 0.006				
Heart rate	− 0.019	P = 0.311					0.027	P = 0.183				
SBP	− 0.173	P < 0.001					− 0.193	P < 0.001	− 0.071	0.025	− 2.40	P = 0.016
DBP	− 0.047	P = 0.115					− 0.131	P < 0.001				
FPG	− 0.075	P = 0.028					− 0.105	P < 0.001				
HDL-c	0.092	P = 0.01					0.081	P = 0.003				
LDL-c	− 0.046	P = 0.119					− 0.037	P = 0.109				
TC	− 0.046	P = 0.121					− 0.07	P = 0.009				
Uric acid	− 0.121	P = 0.001					− 0.14	P < 0.001				
Creatine	− 0.048	P = 0.113					− 0.042	P = 0.077				
eGFR	0.15	P < 0.001					0.009	P = 0.378				

*VAI* visceral adiposity index, *WC* waist circumference, *SBP* systolic blood pressure, *DBP* diastolic blood pressure, *FPG* fasting plasma glucose; *HDL‐c* high‐density lipoprotein cholesterol, *LDL‐c* low‐density lipoprotein cholesterol, *TC* total cholesterol, *eGFR* estimated glomerular filtration rate

**Table 4:** Factors associated with FEV1%predicted by Pearson’s correlation and multivariable stepwise linear regression analysis

	Men						Women					
	Pearson’s analysis		Stepwise linear regression analysis				Pearson’s analysis		Stepwise linear regression analysis			
	Coefficient	P value	β	SE	t	P value	Coefficient	P value	β	SE	t	P value
VAI	− 0.062	P = 0.058					− 0.171	P < 0.001	− 0.097	0.335	− 3.15	P = 0.002
WC	− 0.285	P < 0.001	− 0.287	0.07	− 7.79	P < 0.001	− 0.247	P < 0.001	− 0.217	0.055	− 6.70	P < 0.001
BMI	− 0.192	P < 0.001					− 0.172	P < 0.001				
Age	− 0.206	P < 0.001	− 0.208	0.081	− 5.66	P < 0.001	0.052	P = 0.04	0.139	0.069	4.54	P < 0.001
Heart rate	− 0.024	P = 0.268					0.026	P = 0.193				
SBP	− 0.081	P = 0.02					− 0.118	P < 0.001	− 0.072	0.031	− 2.24	P = 0.025
DBP	− 0.014	P = 0.365					− 0.08	P = 0.004				
FPG	− 0.021	P = 0.294					− 0.071	P = 0.009				
HDL-c	0.053	P = 0.089					0.079	P = 0.004				
LDL-c	− 0.054	P = 0.086					0.038	P = 0.103				
TC	− 0.044	P = 0.132					0.008	P = 0.389				
Uric acid	− 0.105	P = 0.004					− 0.068	P = 0.011				
Creatine	− 0.015	P = 0.353					− 0.033	P = 0.13				
eGFR	0.095	P = 0.008					− 0.001	P = 0.488				

*VAI* visceral adiposity index, *WC* waist circumference, *SBP* systolic blood pressure, *DBP* diastolic blood pressure, *FPG* fasting plasma glucose, *HDL‐c* high‐density lipoprotein cholesterol, *LDL‐c* low‐density lipoprotein cholesterol, *TC* total cholesterol, *eGFR* estimated glomerular filtration rate

The results of the logistic regression analysis for predicting lung function impairment are presented in Table 5.

**Table 5:** The risk of lung function impairment according to each 10% VAI increase

FVC impairment					FEV1 impairment			
	Men		Women		Men		Women	
	OR	P value	OR	P value	OR	P value	OR	P value
Model 1	1.114 (1.008–1.231)	P = 0.034	1.230 (1.147–1.319)	P < 0.001	1.054 (0.952–1.167)	P = 0.313	1.167 (1.082–1.258)	P < 0.001
Model 2	1.168 (1.05–1.299)	P = 0.004	1.218 (1.134–1.307)	P < 0.001	1.097 (0.984–1.222)	P = 0.094	1.160 (1.075–1.252)	P < 0.001
Model 3	1.122 (1.000–1.259)	P = 0.050	1.164 (1.078–1.256)	P < 0.001	1.074 (0.956–1.206)	P = 0.229	1.154 (1.062–1.254)	P = 0.001

Model 1 is unadjusted; Model 2 is adjusted for age, education, smoking and drinking; Model 3 is adjusted for age, education, smoking, drinking, SBP, DBP, FPG, TC, uric acid, and eGFR

*FVC* forced vital capacity, *FEV1* forced expiratory volume in 1 s, *SBP* systolic blood pressure, *DBP* diastolic blood pressure, *FPG* fasting plasma glucose, *TC* total cholesterol, *eGFR* estimated glomerular filtration rate

In the univariate logistic regression model, the ORs for FVC impairment and FEV1 impairment associated with each 10% increase in the VAI were 1.114 (95% CI = 1.008–1.231; P = 0.034) and 1.054 (95% CI = 0.952–1.167; P = 0.313) in men and 1.230 (95% CI = 1.147–1.319; P < 0.001) and 1.167 (95% CI = 1.082–1.258; P < 0.001) in women, respectively. After adjusting for all confounding factors, the adjusted ORs for FVC impairment and FEV1 impairment associated with each 10% increase in VAI were slightly weakened: 1.121 (95% CI = 0.999–1.257; P = 0.052) and 1.081 (95% CI = 0.963–1.213; P = 0.189) in men and 1.161 (95% CI = 1.07876–1.253; P < 0.001) and 1.151 (95% CI = 1.060–1.250; P = 0.001) in women, respectively.

## Discussion

In the present study, we investigated the relationship of the VAI with pulmonary dysfunction among a Chinese middle-aged and elderly population. We found that the VAI value is higher in women than in men, and its relationship with lung function differs slightly between sexes. The VAI is inversely related to FVC%predicted in men and showed a negative association with both FVC% predicted and FEV1%predicted in women. In the linear regression analysis, the decrease in FVC%predicted associated with each 10% increase in the VAI was 1.127% in men and 1.943% in women; the decrease in FEV1%predicted associated with each 10% increase in the VAI was 0.663% in men and 1.738% in women. Further regression analysis revealed that the VAI was positively correlated with FVC impairment and FEV1 impairment in women.

In a previous study, a positive independent relationship was found between lung function impairment and metabolic syndrome, due predominantly to abdominal adiposity [2]. Subsequently, some studies demonstrated that abdominal adiposity can be presented as a marker that permits the early detection of alterations in pulmonary function [3, 5]. WC and BMI are commonly used clinical measures of central obesity, and their association with lung function has been widely demonstrated [6, 21–23]. However, these indicators do not distinguish VAT from subcutaneous adipose tissue (SAT). Recently, VAI has been proposed as a marker of both visceral fat distribution and dysfunction [9]. One way that abdominal obesity may affect lung pulmonary function is through changes in the mechanical properties of the respiratory system. These changes are likely to be due to increased fat surrounding the abdomen, thereby reducing the compliance of the lungs and decreased lung volumes [24]. Visceral fat could also contribute to altering the structure of the diaphragm and restricting diaphragmatic motion, which plays an important role in lung function impairment [25, 26]. In addition, adipose tissue may trigger systemic inflammation because it is an active endocrine organ, the mass of which correlates strongly with the production of proinflammatory cytokines and adipocytokines and negatively with the level of adiponectin [27–29]. Ibrahim et al. clarified that adipocytes present in visceral fat produce more proinflammatory mediators than adipocytes present in subcutaneous fat [30]. Increased levels of serum C-reactive protein (a marker of systemic inflammation) have been positively correlated with decreased lung function [31, 32], obstructive and restrictive lung diseases [33], and visceral fat [29].

Some previous studies have further indicated an inverse relationship between respiratory function and visceral adiposity or fat distribution [4, 34–36]. In 3469 subjects from a Korean cross-sectional study, VAT directly measured using CT was inversely associated with FVC and FEV1 [35]. A similar result was observed in a Japanese population in which abdominal visceral fat was associated with reduced FEV1% predicted, independent of other obesity indices [4]. Another study reported that visceral fat was significantly associated with decreased lung function only in men [36]. However, in a small study involving 40 healthy elderly individuals, pulmonary function was not significantly correlated with MRI-based VAT [7]. This discrepancy may be partly due to the differences in the sample size and characteristics of the participants. Some studies have reported inconsistent results on the relationship between the VAI and obstructive sleep apnea variables [37, 38]. In our study, there was no significant trend in obstructive lung impairment according to VAI quartiles. In addition, it is important to note that, according to our results, there was a sex-related difference between VAI and decreased lung function in that the effect sizes were generally stronger in women.

There are several reports emphasizing the importance of VAT as a metabolic risk factor among women rather than men. A cardiometabolic risk profile was associated with VAT more profoundly among women, and evidence suggested that women may be more sensitive to the inflammatory effects of VAT [41]. In addition, the correlation between VAT and atherosclerosis was stronger in women than in men [42]. Our current study also endorses the importance of VAT on pulmonary function among women. Ethnicity significantly affects abdominal adiposity, and East Asian women have the most deleterious abdominal fat distribution. In comparing VAT and SAT, Park et al. observed that both were important in men, but only VAT was important in women [35]. Importantly, the majority of women in the present study were menopausal and due to changes in circulating sex steroid hormones, the body fat distribution in postmenopausal women tended to switch towards a male pattern. Indeed, menopause is specifically associated with increased central adiposity and insulin resistance. In this sense, a higher value of VAI was observed among women than among men in our study. In addition, women have a higher insulin level than men, yet this finding is not statistically significant (P = 0.052). Moreover, metabolic syndrome has been shown to be a stronger risk factor in women than in men [43]. The increased levels of VAT that often accompany metabolic syndrome correlate with our findings that show increased odds for the impaired lung function associated with VAT in women. Of note, Pearson’s correlation analyses revealed that the association between VAI and lung function is inferior to WC or BMI alone. This may be related to the WC reflecting both SAT and VAT, which contribute to the relationship between abdominal obesity and lung function, respectively. BMI does not distinguish the distribution of body mass or between fat mass and fat-free mass, but it has a strong collinearity with WC and may obtain results similar to those of WC. However, the VAI was regarded as a marker of both visceral fat distribution and dysfunction. The VAI specifically associated with VAT but not with SAT, which was detected by abdominal MRI.

FVC %predicted and FEV1%predicted respectively reflected the maximum ability to for inhalation and forceful expiration in the first second. Because abdominal obesity may affect lung function by changing the mechanical properties of the respiratory system and impeding the space for the lungs to expand, FVC %predicted is the measure that one would expect to be most affected by visceral adiposity accumulation. The results of our current study support this opinion that a higher value of VAI was associated with lower lung volume, with the coefficients of VAI measures being consistently larger when predicting FVC %predicted than FEV1%predicted.

The strengths of our study include the relatively large-scale population-based sample with standardized measures performed following established protocols and stratified by sex. However, this study has some limitations that require consideration. The most important limitation of our study is its cross‐sectional design, and the ability to establish a causal relationship between VAI and lung function impairment is rather limited. Another limitation of the current study is probably the lack of direct data on VAT assessed by MRI or CT. Therefore, we could not further verify the association between VAT and VAI and deduce the role of the VAI in measuring VAT for predicting lung function. In addition, smoking can damage the tracheal mucosa and cause chronic bronchitis-like symptoms, which further affecting lung function. However, due to the limitations of the questionnaire, we did not acquire detailed information about smoking status. In this study, we mainly classified individuals as smokers and nonsmokers. Finally, our studied population was restricted to the middle-aged and elderly population in a rural area in China, so the results should be confirmed in adults and other ethnic populations.

In conclusion, we were the first to report a clear correlation between VAI and lung function impairment. This association was particularly strong among women and was independent of age, education, smoking, alcohol consumption and other metabolic confounding factors. Further large prospective follow-up studies are needed to corroborate our findings. Overall, the VAI may be a simple and useful approach in daily practice, and individuals, especially women with high VAI, should receive additional screening and preventive interventions for respiratory disease.

## Data Availability

The data that support the findings of this study are available from REACTION Study Group but restrictions apply to the availability of these data, which were used under license for the current study, and so are not publicly available. Data are however available from the authors upon reasonable request and with permission of REACTION Study Group.

## Abbreviations

VAI: Visceral adiposity index
VAT: Visceral adipose tissue
SAT: Subcutaneous adipose tissue
WC: Waist circumference
BMI: Body mass index
TGs: Triglycerides
TC: Total cholesterol
HDL-c: High-density lipoprotein cholesterol
LDL-c: Low-density lipoprotein cholesterol
SBP: Systolic blood pressure
DBP: Diastolic blood pressure
FPG: Fasting plasma glucose
HbA1c: Hemoglobin A1c
RIA: Radioimmunoassay
UA: Uric acid
SCr: Serum creatinine
eGFR: Estimated glomerular filtration rate
FVC: Forced vital capacity
FEV1: Forced expiratory volume in 1 s
